# Nipah: The looming post-covid pandemic

**DOI:** 10.6026/973206300200001

**Published:** 2024-01-31

**Authors:** Olivia Sekimoto, Francesco Chiappelli

**Affiliations:** 1 Cajulis Pediatric Dentistry, Chula Vista, CA 91914, USA; 2 Dental Group of Sherman Oaks, Sherman Oaks, CA 91403, USA; 3 UCLA Center for the Health Sciences, Los Angeles, CA 90095, USA

**Keywords:** Nipah Virus - NiV, Nipah viral disease - NiD, SARS-Cov2, CoViD-19, public health, dentists, central nervous system - CNS

## Abstract

First identified as a pathogen in Malaysia and Singapore in 1999, Nipah virus (NiV) caused nearly 300 human cases and over 100 fatalities. It also killed about 1 million pigs. Three years later (2002), it was reported in Pteropus bats in Malaysia, in Cambodia & Thailand, (2005), and as far as Madagascar (2007) and Ghana (2008). India (Kerala) reported its first human NiV-caused fatalities in September 2023. Taken together, these trends emphasize its public health threat. In humans, NiV infection initially leads to fever, headache, body aches and muscle pain, nausea and vomiting. The symptoms rapidly evolve into sore throat, cough and atypical pneumonia leading to severe respiratory distress. The cadre of NiV-induced pathology (Nipah disease, NiD) then includes severe dizziness and drowsiness, progressive alteration in cognition and consciousness, acute encephalitis and seizures. Public health protocols (e.g., mask-wearing, quarantine), essential to contain and control CoViD-19, seem insufficient to contain NiD spread because NiV transmission occurs primarily via direct contacts with body fluids of infected carriers, but presumably not by airborne transmission. As in the case of SARS-C0V2, health care providers (i.e., physicians, dentists, nurses, dental assistants) are greatest risks not only of contracting but of spreading NiV infection. NiV is a high-pathogenicity pathogen, against which, at present, we have no anti-viral medications or preventive vaccine. Taken together, the evidence to date heightens the threat of an upcoming NiD pandemic.

## Nipah Virus (NiV) and Nipah Disease (NiD):

Nipah virus (NiV), a zoonotic high-lethality non-segmented single-strand negative sense RNA virus, uses its homologous RNA-dependent polymerase for replication (i.e., Riboviria). It belongs to the Paramyxoviridae family (e.g., same as measles and respiratory tract infections), the Orthoparamyxovirinae sub-family, and the Henipavirus genus (Nipah Henipavirus species). As other Henipaviruses, NiV is enveloped, and manifests either a filamentous or a spherical structure determined by the N, nucleocapsid, the P phosphoprotein, the M, matrix, the F, fusion, the G, glycoprotein and the L RNA polymerase proteins. P is open-reading frame and encodes for the three nonstructural proteins: C, V and W [1-2]. Despite our knowledge of the molecular biology for NiV, and the fact that NiV infection can be ascertained by serological, molecular, virological or immuno-histochemical diagnostics, there are, at present, no readily available vaccine, anti-viral or effective treatment prophylaxis [1-2]. Though the phylogenetic root of NiV has been traced to 1947, it appears that NiV entered the Southeast-Asian geographical area in two waves (1985, 1995), resulting in two distinct endemic routes:

[1] Traded of infected pigs and domesticated cats and dogs (Malaysia [Kampung Sungai Nipah township, Malaysia] & Singapore), and

[2] Fruit flies bats (genus Pteropus; Malaysia, Cambodia, Thailand, Philippines and eventually as far as Madagascar and Ghana, and more recently southern India) [1-3].

The first wave (1999) caused significant outbreaks of severe encephalitis among pig farmers and abattoir workers in Malaysia with a reported mortality of close to 30%. In toto, comprehensive data between 1998 and 2018 indicate that NiV infection leads to a disease profile (Nipah disease, NiD) with an overall lethality of 40% - 70% of the human cases (https://www.cdc.gov/index.htm). These estimates do not include the recent NiV outbreak with human fatalities in Kerala, India, and Bangladesh. Taken together, these epidemiological data have led to concerted pleas for immediate national (e.g., CDC) and international (i.e., World Health Organization) action [4-5]. The specter of a novel NiD pandemic, arising concurrently with CoViD-19, and potentially compounding the severity and lethality of other current threats to global public health (i.e., SARS-Cov2, SARS-CoV3, Zika virus, Monkeypox Virus) is unquestionable [5,6, 7]. It is timely and critical to identify the most likely mode to control and to counter NiV transmission.

## Preventative measures against NiV:

In contrast to the last five pandemics, which have been initiated in the pharyngo-oro-nasal structures [8], NiV transmission does not appear to occur by airborne transmission, but through consumption of infected foods or contact with human bodily fluids [9]. Indeed, evidence to date indicates that NiV transmission derives from close contact with infectious bodily secretions (i.e., saliva, sputum, nasal lavages, sweat) from clinically NiV-positive patients [8-9].

Traditional public health measures (e.g., masks, gloves, quarantine) may therefore be less effective in containing an emerging NiD pandemic than they proved to be for CoViD-19. Nonetheless, the use of personal protection equipment and good hygiene practices are recommended prevention methods for person-to-person NiV transmission. Isolation and surveillance of infected patients is necessary until they test negative. NiV fomites can be inactivated from surfaces by soaps, detergents, or disinfectants like sodium hypochlorite [10].

## Pathological Course of NiD:

In humans, NiV is responsible for a progressive illness, which affects the respiratory system and the central nervous system (CNS), and eventually multiple organs. Entry of the virus can occur through inhalation or ingestion of infected material. NiD pathological course can be outlined as follows:

[1] As any other viral infections, NiV initially causes mild discomfort (e.g., fever, headache, body aches, nausea), which soon develops into severe pneumonia and respiratory impairment, as NiV infects the epithelial cells of the bronchiole and pneumocytes that promote airway stability. NiV replicative cycle occurs then in the respiratory epithelium, and NiV is shed througout the respiratory tract. Acute pneumonia, potentially lethal respiratory distress ensue [11].

[2] NiV can target the CNS either via the olfactory nerve (Cranial nerve I). When the olfactory epithelium is infected by NiV, the virus can spread throughout the ventral cortex and olfactory tubercle, and enter the CNS. Brain infections by NiV leads to dizziness and drowsiness, alteration in cognition and consciousness, seizures, encephalitis and ultimately coma and death [12].

[3] NiV can also enter the bloodstream, and contribute to systemic vasculitis with extensive thrombosis and parenchymal necrosis, endothelial cell damage, cellular necrosis and syncytial giant cell formation potentially in a variety of target, leading to multiple organ damage and failure [13].

## Oral Immunity:

In conclusion and as noted, NiV transmission occurs through contact with saliva and other bodily fluids of infected patients. Thus, certain segments of the human populations are at greater risk of NiV because of exposure to body fluids. Namely, physicians, dentists, nurses, dental assistants and dental patients can not only contract but spread NiV infection. Saliva droplets that are generated by speech, coughing or sneezing produce a silent spread of viral particles from asymptomatic patients. Respiratory RNA viruses, including NiV, primarily infect and replicate in the respiratory tracts and shed into the oropharyngeal passage, bronchi and the lungs. This pattern for infection was evinced in previous pandemics, from the 1917/8 Spanish Flu to the 2009 Swine Flu, and the current CoViD-19 [9-11].

In the Fourth and current Industrial Revolution, natural landscapes, farmlands and ecosystems continue to be destroyed due to human activity. The gradual warming of global temperatures induces the thawing of peri-glacial permafrost which can release ancient and novel pathogens into the atmosphere. As we noted elsewhere [14-15], RNA and giant viruses are among some of the permafrost pathogens that are released and can potentially be harmful to our immune system. It is therefore timely and critical to understand NiV and its effects on our oropharyngeal immunity and respiratory systems to be better equipped to confront novel pathogens that may arise with melting permafrost.

Compartmentalized oro-pharyngeal immunity against NiV commences at the oral mucosa, and is mediated in part by the dendritic cells, macrophages, neutrophils and B cells, and largely regulated by the resident cytotoxic T cells (CD3+CD8+CD38+). These T cells will engage activation of tissue-resident memory CD8CD45R0 T cells (CD8RM) cells at the site of viral replication. As it is the case for other pathogens of the oral cavity, NiV antigen-specific CD8+ T cells come to constitute most of the early circulating pool of ant-NiV effector CD8+ T cell (CD8EFF) that enter the lung to proffer immunity to the respiratory tract [16- 17]. A similar compartmentalized nasal mucosa immune process will provide initial immune protection to the olfactory epithelium against NiV and other respiratory viruses, perhaps preventing NiV penetration into the CNS.

Taken together, our knowledge base of mucosal viral immunity and NiV pathology should help in the development of effective anti-NiV vaccine strategies. The elucidation of fundamental molecular facets of Riboviria in general, and more specifically of the Henipavirus genus will contribute to the development, testing and evaluation of urgently needed specific anti-NiV anti-viral agents.
